# Impulse buying behavior during livestreaming: Moderating effects of scarcity persuasion and price perception

**DOI:** 10.1016/j.heliyon.2024.e28347

**Published:** 2024-03-18

**Authors:** Zhitan Feng, Abdullah Al Mamun, Mohammad Masukujjaman, Mengling Wu, Qing Yang

**Affiliations:** a School of Art and Media, Nantong Open University, Nantong City, Jiangsu Province, 226000, China; b UKM - Graduate School of Business, Universiti Kebangsaan Malaysia, 43600, UKM Bangi, Selangor Darul Ehsan, Malaysia; c Faculty of Business Management and Professional Studies, Management and Science University, 40100, Shah Alam, Selangor, Malaysia

**Keywords:** Livestreaming, Online impulsive buying, Price perceptions, S–*O*-R theory, Susceptibility to social influence, Scarcity persuasion

## Abstract

This research aimed to identify the factors that influence impulse buying behavior during livestreaming and advance the existing literature based on a proposed conceptual framework grounded in the stimulus-organism-response (S–*O*-R) model. We also tested the moderating effects of price perception and scarcity persuasion. An online self-administered questionnaire was used to collect data from 837 Chinese participants aged over 18 years. The data were analyzed using partial least squares structural equation modeling using Smart-PLS version 4.0. The findings showed that susceptibility to social influence, impulse buying tendency, cognitive reactions, affective reactions, and the urge to buy impulsively are statistically significant predictors of impulse buying during livestreaming, with price perception and scarcity persuasion as moderators. The study expands the S–*O*-R model for livestreaming impulse buying in e-commerce context, highlighting its multifaceted nature and revealing the mediating role of Urge to Buy Impulsively in translating cognitive and emotional factors into impulse buying behavior. These insights offer practical guidance for marketers to design tailored strategies that leverage psychological triggers and external cues to enhance consumer engagement and encourage desired behaviors, ultimately leading to more effective marketing campaigns and improved consumer experiences.

## 1 Introduction

In the era of digital transformation, e-commerce has reshaped consumer behavior, offering unprecedented convenience [1]. The emergence of livestreaming in online retail has transformed the shopping experience, allowing real-time interaction and unique engagement [2]. Livestreaming's distinct features, like vendor-customer interaction and virtual gifts, make it interactive and exciting [[3], [4], [5], [6]]. Impulse buying during livestreaming is a fascinating phenomenon [7]. It's characterized by unplanned emotional purchases and presents both challenges and opportunities [8]. Livestreaming is gaining immense popularity in China, enhancing selling efficiency and the buying experience [9]. Nearly 40% of Chinese internet users are involved in livestreaming [10]. In 2021, livestreaming e-commerce generated 2.36 trillion yuan, and it's projected to grow significantly, reaching over USD 534.37 billion [11]. The Chinese e-commerce industry is booming, set to grow by US$720 trillion in 2023, accounting for 15% of all e-commerce [12]. Major players in China include Taobao.com, JD.com, and Pingduoduo.com [7], showcasing the massive growth potential of livestreaming e-commerce in China.

This phenomenal growth of livestreaming e-commerce in China is gaining scholarly interest; however, the research is in its infancy, as the phenomenon has only developed in China over the last five years [5]. The burgeoning popularity of livestreaming platforms, coupled with the rapid growth of e-commerce, highlights the necessity of understanding the complex interplay of predictor that affect consumer behavior in this evolving landscape. Livestreaming provides an avenue for businesses to create a sense of community, allowing viewers to interact with hosts, ask questions, and receive real-time responses [4]. This interpersonal element adds a layer of social influence that can stimulate impulse buying decisions in novel ways. Additionally, livestreaming offers a sense of authenticity and transparency as hosts showcase products in real-time, potentially addressing the lack of tangibility that often deters online shoppers. Moreover, developing a deeper understanding of the determinants of impulse purchase decisions among Chinese consumers during livestreaming is significantly crucial for business leaders and new entrants to facilitate better livestreaming e-commerce platforms for Chinese consumers.

There has been a significant shift in impulsive buying behavior compared to Stern's original proposal [13], particularly in the context of brick-and-mortar stores. When Stern introduced his ideas, online shopping was not as prevalent as it is today. Stern pointed out that the convenience of purchasing and the accessibility of products would likely lead to more impulsive buying decisions. Additionally, consumer behaviors have evolved significantly over the years due to increased involvement in purchase decisions, changes in income, spending habits, age demographics, purchasing capabilities, and a shift from lifestyle-based to experience-based buying [14]. Consequently, Stern's original model has incorporated various new aspects [15]. Several factors, including external stimuli and internal factors, play a crucial role in making online purchase decisions. This necessitates the development of new theories capable of accurately predicting online impulse buying.

In the realm of online purchasing, the Stimulus-Organism-Response (S–*O*-R) theory has proven to be a suitable theoretical framework for understanding how web-based stimuli impact online consumer behavior [16,17]. While the S–*O*-R theory has traditionally been applied to in-store shopping, it is also being utilized in the context of online shopping. Within this theory, a stimulus represents an external factor that triggers internal organismic states, as delineated by Mehrabian and Russell [18]. Although some previous studies on online shopping [6,19,20] and other studies on impulse buying [16,17] have adopted the S–*O*-R theory, these studies are not comprehensive. They have paid limited attention to the livestreaming context and have overlooked important psychological factors such as cognitive reactions (CR), affective reactions (AR), and individual impulse buying tendencies (IIB). While Lo et al. [20] integrated these constructs into livestreaming e-commerce, they did not elucidate the causal and consequential processes involved in the adoption decision using the S–*O*-R theory. A few studies, like Ming et al. [7], have considered the S–*O*-R theory in the context of livestreaming but have somewhat neglected impulsive buying intention as a crucial predictor of impulsive buying behavior.

Similarly, some research has been conducted on livestreaming shopping in China; however, much of it has examined the effects of convenience, interactivity, playfulness [21], celebrity endorsements [22], interface design, live atmosphere [6,19], human–computer interaction [19], and website attributes [23]. Surprisingly, with the exception of a few studies, the influence of susceptibility of social influence, affective reactions, cognitive reactions, and individual impulse buying tendency toward IBB has received very limited attention, with the exception of a few studies [[24], [25], [26]] demonstrating the significant impact of susceptibility to social influence, affective reactions, cognitive reactions, individual IIIB, and the UBI on predicting individual IIB. These critical factors cannot be ignored, as they have shown statistical significance in previous research. Therefore, the existing limited knowledge on IBB and livestreaming needs to be advanced.

Thus, to fill this gap in the existing literature, the current research aims to examine the effects of susceptibility of social influence, affective reactions, individual impulse buying tendency, cognitive reactions, and UBI on IBB while testing the moderating effects of scarcity persuasion and price perception toward impulse buying behavior during livestreaming. By addressing such gap in the prevailing knowledge, the study delves into providing valuable insights for businesses, marketers, and researchers alike. Understanding the nuances of impulse buying during livestreaming can enable businesses to design more effective strategies, tailor content to resonate with viewers, and foster a deeper connection between hosts and consumers. Moreover, the research contributes to the broader academic discourse on consumer behavior by extending our understanding of how digital environments reshape traditional concepts. As the world continues its shift towards a digitally driven economy, the exploration of impulse buying behavior during livestreaming holds not only theoretical significance but also practical implications for businesses seeking to navigate the ever-evolving landscape of online retail. In particular, this research significantly advances the S–*O*-R theory to the Chinese context by incorporating scarcity persuasion, price perception, susceptibility to social influence, individual impulse buying tendency, cognitive reactions, and affective reactions, shedding light on previously overlooked variables. The study will demonstrate the multifaceted nature of impulsive buying behavior, highlighting the complex interplay of psychological and emotional factors driving consumer inclination towards impulsive purchases. Additionally, it provides deeper insights into impulse buying within livestreaming e-commerce by considering scarcity persuasion and price perception as moderators, particularly in the Chinese context, enhancing our understanding of external influences. Lastly, the study will reveal the mediating effect of the urge to buy impulsively on the relationships between various psychological and emotional factors and impulsive buying behavior, contributing to a more nuanced comprehension of its cognitive processes.

## 2 Theoretical foundation and hypothesis development

### 2.1 Stimulus–organism–response model

The S–*O*-R model has been widely applied, particularly in relation to impulse buying. For instance, Ming et al. [7] used the S–*O*-R model to test the effect of social presence on impulse buying during livestreaming. Khoi and Le [27] incorporated the model to test convenience and interactivity as stimuli and impulse buying as a response during livestreaming. Yu et al. [28] investigated perceived professionalism, gamification, and telepresence as stimuli and innovativeness as the organism to lead individuals to impulse buying as a response. Li et al. [29] integrated the S–*O*-R model using broadcasters’ social presence and livestreaming through pleasure and arousal as organisms leading to impulse buying as a response. Thus, the S–*O*-R model is arguably one of the most suitable to examine consumer behavior in relation to impulse buying during a livestream. This model provides a better and logical explanation how individual takes decision based on external and internal factors to reach a decision based on the clear indication of causes and effects. Therefore, we adopted this model in our study.

In our research, the external factors like scarcity persuasion, and price perception is incorporated as the stimulus component, while susceptibility to social influence [30], individual impulse buying tendency [31] cognitive reactions [32], and affective reactions [33], are the organism, whereas UBI [34] and impulse buying behavior are the response [29]. The conceptual framework of this research was established by incorporating constructs from prior studies on online impulse buying.

### 2.2 Urge to buy impulsively

The urge to buy impulsively is a state of having a desire which is experienced upon being exposed to an object in a particular environment [35]. In this study, the urge to buy impulsively refers to an online consumer's sudden and spontaneous desire to buy when watching a livestream. Lou and Yuan [36] argued that several determinants lead to an UBI, such as social influence. Lee and Chen [37] proposed that affective reactions (e.g., perceived enjoyment) have a statistically significant effect on the UBI. Buyers are heavily exposed to information and engagement on social platforms that influence their desire to make impulse purchases as online shopping rapidly transforms into social commerce [34]. Further, the UBI is also the prior intention of the customers of actual impulse purchase [38]. Some studies also proposed that the UBI is the key component of actually making an impulse purchase [39]. Past studies have focused on e-commerce [40] and social commerce [41]. However, with the popularity of livestreaming platforms, many merchants now use them to sell products and find ways to guide customers to make impulse purchases, and livestreaming e-commerce has appeared as a new trend [37]. Therefore, this study focused on impulse buying during livestreaming.

### 2.3 Susceptibility to social influence and the urge to buy impulsively

Susceptibility to social influence can be defined as being easily persuaded by what other people experience and desiring their approval [42]. Scheinbaum et al. [43] posited that this influence varies, as some individuals are more susceptible to being influenced by others when making purchase decisions. Thus, people who are more SSI may exhibit a greater likelihood to depend on others to confirm their purchase decisions [44]. Previous studies have established a strong positive relationship between SSI and the UBI [24]. Sharma and Klein [30] found that SSI has a significant impact on online purchasing, which was further supported by Polas et al. [45], who identified a positive relationship between SSI and impulse buying. Hence, the preceding argument gives rise to the subsequent hypothesis.

H1 : Susceptibility to social influence is positively connected to the urge to buy impulsively.

### 2.4 Impulse buying tendency and the urge to buy impulsively

Impulsive buying tendency denotes the degree to which a person will make immediate and unplanned purchase decisions [35]. Utama et al. [46] noted that each individual has different level of impulse buying tendency, and people with higher tendencies often have a strong urge to make immediate purchases. As a personal trait, a higher impulse buying tendency will help stimulate a stronger impulse buying desire. Bellini and Aoilfi [47] posited that people with stronger impulse buying tendencies experience higher urges to impulsively make purchases. Previous studies [25,[48], [49], [50]] have indicated that individual IIB and urge to buy impulsively are strongly and positively connected. Bandyopadhyay [51] argued that differential tendencies to exhibit IBB will drive different levels of the UBI. Cavazos-Arroyo & Máynez-Guaderrama [31] further validated Bandyopadhyay's [51] findings, claiming that consumers high impulse purchase tendencies are more likely to eventually make a purchase without a second thought. Aiolfi et al. [50] similarly reported that several factors play major roles in driving consumers' UBI, and IIB showed that greater effect. As a result of the aforementioned reasoning, the following hypothesis emerges.

H2 : Individual impulse buying tendency is positively associated to the urge to buy impulsively.

### 2.5 Cognitive reactions and the urge to buy impulsively

Cognitive reactions are the mental processes individuals experience as a result of being exposed to a stimulus [52]. Individuals will cognitively evaluate received information, and positive cognitive processes will enhance consumers’ impulse buying desires for specific products, services, or brands [20]. Cognitive reactions have shown a positive and significant relationship with impulse buying in previous studies [53]. Zuo and Xiao [53] noted that cognitive reactions lead to individuals being more likely to make instant buying. Wu et al. [32] demonstrated that cognitive reactions are significant triggers for impulse purchases. Cui et al. [54] noted that perceived usefulness is one variable of cognitive reactions and reported a significant positive link with the UBI. Paul et al. [55] investigated CR (e.g., perceived usefulness) and further validated the significant positive link between CR and the UBI. Hence, based on the argument above, the hypothesis was postulated below.

H3 : Cognitive reactions are positively linked to the urge to buy impulsively

### 2.6 Affective reactions and the urge to buy impulsively

Affective reactions refer to the arousal, pleasure, and dominance of affective responses to an external environmental trigger [56]. Perceived enjoyment is among the main determinants of impulse purchasing and the most commonly utilized variable of affective reactions [37]. Previous studies [40,57,58] have found that affective reactions have significant positive effects on the UBI. Zuo and Xiao [53] asserted that livestreaming shoppers' perceived enjoyment has a considerable favorable impact on their propensity toward making impulse purchases. Zhang et al. [26] studied the effects of affective reaction on impulsive online behavior and found a strong positive relationship. Parboteeah et al. [52] reported the similar findings on the connection between affective reactions and the UBI. Yang et al. [19] presented similar findings, reporting that the urge to buy impulsively is determined by consumers' affective reactions. Chen et al. [33] stated that, apart from the general gratification experienced in online shopping, intimacy and personal closeness have a role in establishing an urge to buy. Chen et al.‘s [33] findings were in line with those of Xiang et al. [40], who reported a close positive relationship between AR and the UBI. Similarly, Tang and Meng [59] found that affective reactions are statistically significantly connected with the UBI. Consequently, deriving from the aforementioned reasoning, the subsequent hypothesis was postulated.

H4 : Affective reactions is positively linked to the urge to buy impulsively.

### 2.7 Urge to buy impulsively and impulse buying behavior

Impulse buying refers to making an unplanned hedonic and compelling purchase without deliberately analyzing relevant factors and alternatives [52]. Akram et al. [8] proposed that the phenomena of impulse buying occur strong emotions lead to people making purchases without prior planning. Bandyopadhyay et al. [39] noted that the UBI is a significant factor of IBB. Similarly, Zhang et al. [26] argued that the UBI is a stage prior to when consumers actually engage in IBB, indicating that the UBI has the ability to predict IBB. Cavazos-Arroyao et al.‘s [31] findings were in consistent with those of Zhang et al. [26], as they found that the UBI and IBB are strongly and closely related. Chen et al. [60] reported statistically significant findings that the UBI leads individuals to IBB. Similarly, Aiolfi et al. [50] stated that a higher UBI translates to individual IBB. Therefore, we put forth the following hypothesis.

H5 : The urge to buy impulsively is connected to impulse buying behavior.

### 2.8 Mediating effect of the urge to buy impulsively

The urge to buy impulsively is a crucial variable in driving online consumers to actually make an impulse purchase [46], because when consumers have an immediate desire to buy something, they are less likely to withstand the urge. A sudden urge can lead to an impulse purchase based on the following factors: susceptibility to social influence [30], affective reactions [26], cognitive reactions [53], individual impulsive buying tendency [31], and cognitive reactions [24]. Susceptibility to social influence is a significant construct in creating an immediate need to purchase a particular service or product. Goel et al. [61] and Zafar et al. [41] noted that people with impulsive buying tendencies are prone to exercise low conscientiousness, have weak affective autonomy, and are more action oriented, which leads to the development of a stronger desire to act impulsively. Regarding cognitive reactions, Cui et al. [54] considered usefulness to be a relevant variable, and their findings showed a favorable and substantial link with the tendency to make impulse purchases. Thus, cognitive reactions have been found to have a statistically significant connection with an instant urge to buy [32]. Regarding affective reactions, consumers will have the urge to make an immediate purchase if they perceive online shopping to be a more enjoyable experience. According to Zuo and Xiao [53], livestreaming customers’ opinions on how much fun they are having influences the likelihood consumers will make impulsive purchases. Chen et al. [33] noted that personal closeness and intimacy are also factor in establishing purchase desire, aside from the general gratification associated with online shopping. Thus, the following hypotheses were developed:

H6–9: The urge to buy impulsively mediates the effects of susceptibility to social influence, impulse buying tendency, cognitive reactions, and affective reactions on impulse buying behavior.

### 2.9 Moderating effect of scarcity persuasion

Scarcity sends a signal to consumers that a product's popularity exceeds its supply, thereby indicating its value, quality, or exclusivity, and leading consumers to perceive a product as scarce stimulates purchase behavior [62]. In this research, scarcity persuasion is denoted as the extent to which consumers perceive being persuaded to buy goods, services, or resources that are difficult to access [4]. Buying intention is sometimes triggered by product scarcity, as some previous marketing-related research has demonstrated [11,63]. Chung et al. [64] also found that scarcity will encourage customer to make impulse purchases. Park et al. [65] noted that scarcity is a significant moderator in strengthening the relationship between consumers' attitudes toward luxury products and their willing to pay. Therefore, scarcity persuasion could play a positive role in increasing the likelihood consumers will make impulse purchases during livestreams. Drawing from the preceding discussion, the subsequent hypothesis was formulated.

H10 : Scarcity persuasion moderates the link between the urge to buy impulsively and impulse buying behavior.

### 2.10 Moderating effect of price perception

Price perception is referred to as the value of the money and sacrifices consumers give to obtain a product [66]. Lichtenstein et al. [67] proposed that the cost to consumers to obtain a product could result in a positive or negative signal for behavior, because some customers perceive a higher price as representing spending more economic resources, while others believe that a high price represents a high-quality product. Therefore, price perception will affect customers’ impulse buying process. Further, this effect is even more pronounced in online shopping, as users can compare prices with less cost [68]. According to Sarkar and Khare [69], price perception plays a key role in online purchases. Bose and Banerjee [70] found that price perception moderately affects purchase intention. Fenneman et al. [71] also reported that price perception has a moderating role in online shopping, while Muge [72] found price perception to have statistically negative moderating effects. Thus, we present the subsequent hypothesis.

H11 : Price perception moderates the connection between the urge to buy impulsively and impulse buying behavior.

## 3 Research Methodology

### 3.1 Sample and population

This study mainly focused on online impulse buying during livestreaming. The target population comprised Chinese online shoppers who had experience watching livestreams. This study utilized a non-probability convenience sampling method due to the absence of a sampling frame and the unknown population size. G*Power software was to obtain a minimum representative sample size. According to Faul et al. [73] the sample size generated by G*Power can be used to support the sample size and power calculation of various statistical tests. To examine seven variables, with a power of 0.95 and an effect size of 0.15, G*Power calculated the minimum sample size to be 153. Through using an online survey for data collection, this study ultimately obtained 837 valid respondents. However, it is worth noting that Hair et al. [74] recommends a minimum sample size of 200 for PLS-SEM analyses.

### 3.2 Data collection method

This study incorporated a deductive and quantitative research method. The data were collected using a cross-sectional research design in which each participant took part in the study at one time. Data were gathered in China through an online survey. To ensure participants could understand the questionnaire, a pilot survey was conducted with a population that was 10% of the total sample size. Then, final data collection was performed using a questionnaire link generated WJX, which is a well-known online survey platform. The link was distributed through popular social media platforms, such as QQ and WeChat. In addition, respondents were informed that the questions were related to their personal opinions, without any right or wrong answers.

In our research study, participants willingly and knowingly provided their consent before participating. They were provided with comprehensive details about the study's aims, data usage, and the option to withdraw at any point without facing adverse consequences. In order to maintain confidentiality, stringent protocols were implemented to manage the collected data. Any personal identifiers were either eliminated or anonymized to protect participants' anonymity. The data were securely stored and limited to authorized researchers only. The collected information was exclusively used for academic and research purposes pertaining to this article. We took special care to aggregate the data to ensure the confidentiality of individual participants. The study was initiated following ethical approval. Throughout the research, strict adherence to approved ethical guidelines and relevant regulations was maintained.

### 3.3 Instruments

A self-administered survey questionnaire was prepared that measured the constructs related to impulse buying behavior during livestreaming. The proposed variables were adapted from the literature on impulse buying during livestreaming to suit the context of this study. The questionnaire comprised two main sections. The first section collected respondents’ demographic information, such as age, gender, and occupation, and included other questions related to livestreaming online shopping. The second section included items related to each construct proposed in the study. The items on susceptibility to social influence were adapted from Bearden et al. [42], Bai et al. [75], and Le [76]; the measurement of individual impulse buying tendency was from Madhu et al. [77]; the items on cognitive reactions were from Ding et al. [78]; the items on affective reactions were adapted from Malafe et al. [79] and Lo et al. [20], the instrument used to assess the UBI was adapted from Rook and Fisher [80], Kacen and Lee [81], Xiang et al. [40], Herzallah et al. [82], and Goel et al. [61]; the items on scarcity persuasion were adapted from Wu et al. [83] and Wu et al. [84]; the items on price perception were adapted from Park et al. [85] and Do et al. [86]; and the items on IBB were adapted from Goel et al. [61] and Tran and Nguyen [87]. A seven-point Likert scale, spanning from “strongly disagree” to “strongly agree,” was employed to measure all the items. Prior to distributing the questionnaires, experts translated them into Mandarin from the original English versions. The questionnaire is shown in Supporting Material - ***S1*.** *Survey Instrument*. Complete data submitted with the manuscript as Supporting *Material - S2. Survey Dataset.*

### 3.4 Common method bias

When participants simultaneously respond to pre-established questions, common method bias (CMB) may be present in a study [88]. Thus, this study adopted two remedies to control and examine the issue. One was Harman's one factor test, which indicated that there was no issue with CMB, as the total variance extracted by one factor was 34.41%, which was significantly less than the threshold of 50%. The other was a full collinearity test, the findings of which showed that the variance inflation factor (VIF) values for each variable were less than 3.3, indicating that no CMB was present [89]. The details are shown in Table 1.

**Table 1:** Kock's FCT – VIF values.

Variables	SSI	IIB	CR	AR	UBI	SP	PP	IBB
VIF	1.517	1.515	1.504	1.429	2.166	1.549	1.514	1.674

**Notes:** SSI: Susceptibility to Social Influences; IIB: Impulse Buying Tendency; CR: Cognitive Reactions; AR: Affective Reactions; UBI: Urge to Buy Impulsively; SP: Scarcity Persuasion; PP: Price Perception; IBB: Impulse Buying Behavior.

**Source:** Author's data analysis.

### 3.5 Multivariate normality

The Web Power tool was used to assess the multivariate normality, which can calculate the significant values of skewness and kurtosis. The test generated a p-value <0.05, indicating the existence of multivariate non-normality [90].

### 3.6 Data analysis method

The proposed model in this study was tested using the PLS-SEM method and included two stages [74]. The first stage was the evaluation of the measurement model, and the validity and reliability of the constructs were assessed in this stage [74]. The second stage was the structural model evaluation, which aimed to assess the size of the associations between the constructs related to impulse purchasing behavior. PLS-SEM is a of preferred method in market and management research because it can estimate statistical models containing a wealth of data, which is essential when researchers are looking for probable causes for a variety of variables [74]. Many researchers [29], particularly in the field of online impulse buying, have used PLS-SEM. Furthermore, the multivariate normality test in this research suggested non-normality in the data distribution, leading us to adopt PLS-SEM as recommended by Hair et al. [74]. Besides, PLS-SEM offers several advantages over other methods like ‘Total Interpretive Structural Modeling’ and ‘Fuzzy Decision-Making Trial and Evaluation Laboratory’. Firstly, PLS-SEM is well-suited for complex models with small sample sizes, making it more applicable to research scenarios where data may be limited or non-normal [74]. Secondly, PLS-SEM allows for simultaneous estimation of measurement and structural models, providing researchers with a comprehensive understanding of both the relationships between variables and their measurement reliability [74]. Additionally, PLS-SEM is robust to multicollinearity issues, making it particularly useful when dealing with highly correlated predictor variables. Lastly, PLS-SEM is flexible in handling reflective and formative constructs, accommodating different types of latent variable measurement, which enhances its versatility in various research contexts [74]. Considering the advantages outlined above, PLS-SEM was chosen over the aforementioned methods.

## 4 Results

### 4.1 Sample characteristics

Table 2 shows the respondents' demographic information. The majority of the participants were women (61.2%). The largest proportion of participants (28.6%) were between 26 and 35 years old, and the smallest proportion were above 65 years old (2.5%). Approximately half of the participants (50.2%) were married, followed by single participants (43.5%). Most participants had a bachelor's degree, accounting for 55.9% of the total. Further, nearly half of the respondents (44.8%) had full-time employment, and only 1% were unemployed. All the participants reported having experiencing watching livestreams. Regarding viewing frequency, 30.6% of the participant viewed livestreams daily, 24% often viewed then and 28.8% often watched them, and only 4.5% participant rarely watched them. For average monthly income, 23.2% participants earned between RMB3001 to RMB4500; however, only 6.3% earned above RMB7500. Regarding livestreaming shopping frequency, most participants (56.5%) shopped one to three times per month, while 7.8% shopped more than eight times per month. More than three-fifths of the respondents spent below RMB1500 monthly, and only a few respondents spent above RMB7500.

**Table 2:** Demographic profile of respondents.

	n	%		n	%
*Gender*			*Average monthly income*		
Male	325	38.8	Less than RMB1500	146	17.4
Female	512	61.2	RMB1500–RMB3000	182	21.7
Total	837	100.0	RMB3001–RMB4500	194	23.2
			RM4501–RMB6000	134	16.0
*Age*			RMB6001-RMB7500	128	15.3
18–25 years	202	24.1	More than RMB7500	53	6.3
26–35 years	239	28.6	Total	837	100.0
36–45 years	204	24.4			
46–55 years	127	15.2	*Watching time*		
56–65 years	44	5.3	Less than 1 month	71	8.5
Above 65 years	21	2.5	1–3 months	146	17.4
Total	837	100.0	3–6 months	216	25.8
			6 months to 1 year	163	19.5
*Marital status*			1–2 years	153	18.3
Single	364	43.5	More than 2 years	88	10.5
Married	420	50.2	Total	837	100.0
Divorced	41	4.9			
Widowed	12	1.4	*Viewing frequency*		
Total	837	100.0	Everyday	256	30.6
			Often	201	24.0
*Education level*			Always	241	28.8
College degree and below	246	29.4	Sometimes	101	12.1
Bachelor's degree	468	55.9	Rarely	38	4.5
Master's degree	105	12.5	Total	837	100.0
Doctorate degree	18	2.2			
Total	837	100.0	*Livestreaming shopping frequency*		
			1–3 times per month	473	56.5
*Employment status*			4–7 times per month	299	35.7
Employed full time	375	44.8	More than 8 times per month	65	7.8
Employed part time	90	10.8	Total	837	100.0
Self-employed	163	19.5			
Student	179	21.4	*Monthly spending on product shopping*		
Unemployed	8	1.0	Less than RMB1500	510	60.9
Retired	22	2.6	RMB1500–RMB3000	177	21.1
Total	837	100.0	RMB3001–RMB4500	100	12.0
			RMB4501–RMB6000	35	4.2
*Watch livestreams*			RMB6001–RMB7500	10	1.2
Yes	837	100.0	More than RMB 7500	5	0.6
Total	837	100.0	Total	837	100.0

**Note:** 1 USD = 6.95 RMB.

### 4.2 Validity and reliability

This study tested for internal consistency, convergent validity, and discriminant validity to ensure the validity and reliability of the measurement model. The internal consistency of the constructs was measured using Cronbach's alpha, composite reliability, and reliability coefficient ρ_A_. Thus, as shown in Table 3, the values for Cronbach's alpha, composite reliability, and reliability coefficient ρ_A_ for each construct were above the threshold of 0.7, confirming the internal consistency of all constructs. Average variance extracted (AVE) was evaluated to test convergent validity. The results in Table 3 show that convergent validity was established, as the AVE values were all above the threshold of 0.50. The Fornell-Larcker criterion, cross-loadings, and the and heterotrait-monotrait ratio (HTMT) were assessed to determine the discriminant validity. The Fornell-Larcker values in Table 4 show that, for each column, the AVE square roots are larger than the internal determinant correlations. Table 5 indicates the factor loadings of each indicator. Kline [91] recommended the acceptable HTMT threshold of below 0.85, and the results in Table 4 show that the HTMT values were all below this threshold. Thus, based on all these three assessment approaches, the study successfully demonstrated the distinctiveness of the constructs' validity through discriminant analysis.

**Table 3:** Reliability and validity.

Variables	Items	Cronbach's alpha	rho_A	Composite reliability	Average variance extracted	Variance inflation factor
SSI	5	0.865	0.865	0.903	0.650	1.367
IIB	5	0.897	0.898	0.924	0.708	1.321
CR	5	0.876	0.876	0.909	0.668	1.385
AR	5	0.918	0.922	0.938	0.753	1.256
UBI	5	0.914	0.915	0.936	0.744	1.330
SP	5	0.905	0.906	0.929	0.725	1.687
PP	5	0.907	0.909	0.930	0.728	1.681
IBB	5	0.907	0.907	0.931	0.728	–

**Notes:** SSI: Susceptibility to Social Influence; IIB: Impulse Buying Tendency; CR: Cognitive Reactions; AR: Affective Reactions; UBI: Urge to Buy Impulsively; SP: Scarcity Persuasion; PP: Price Perception; IBB: Impulse Buying Behavior.

**Source:** Author's data analysis.

**Table 4:** Discriminant validity.

	SSI	IIB	CR	AR	UBI	SP	PP	IBB
*Fornell-Larcker criterion*								
SSI	0.806							
IIB	0.405	0.841						
CR	0.413	0.405	0.817					
AR	0.359	0.306	0.377	0.868				
UBI	0.515	0.531	0.502	0.506	0.863			
SP	0.350	0.347	0.346	0.337	0.431	0.851		
PP	0.372	0.344	0.390	0.341	0.420	0.463	0.853	
IBB	0.398	0.407	0.343	0.286	0.522	0.497	0.449	0.853
*Heterotrait-monotrait ratio (HTMT) - Matrix*								
SSI								
IIB	0.459							
CR	0.474	0.455						
AR	0.402	0.335	0.420					
UBI	0.578	0.585	0.560	0.549				
SP	0.395	0.385	0.388	0.370	0.473			
PP	0.422	0.382	0.437	0.374	0.460	0.511		
IBB	0.449	0.450	0.385	0.312	0.572	0.547	0.493	

**Notes:** SSI: Susceptibility to Social Influences; IIB: Impulse Buying Tendency; CR: Cognitive Reactions; AR: Affective Reactions; UBI: Urge to Buy Impulsively; SP: Scarcity Persuasion; PP: Price Perception; IBB: Impulse Buying Behavior.

**Source:** Author's data analysis.

**Table 5:** Factor loadings.

	SSI	IIB	CR	AR	UBI	SP	PP	IBB
SSI1	0.789							
SSI2	0.798							
SSI3	0.836							
SSI4	0.813							
SSI5	0.792							
IIB1		0.820						
IIB2		0.850						
IIB3		0.869						
IIB4		0.846						
IIB5		0.822						
CR1			0.807					
CR2			0.809					
CR3			0.817					
CR4			0.823					
CR5			0.829					
AR1				0.884				
AR2				0.882				
AR3				0.868				
AR4				0.850				
AR5				0.856				
UBI1					0.868			
UBI2					0.872			
UBI3					0.888			
UBI4					0.841			
UBI5					0.844			
SP1						0.846		
SP2						0.852		
SP3						0.868		
SP4						0.847		
SP5						0.844		
PP1							0.851	
PP2							0.836	
PP3							0.860	
PP4							0.849	
PP5							0.870	
IBB1								0.844
IBB2								0.857
IBB3								0.864
IBB4								0.866
IBB5								0.836

**Notes:** SSI: Susceptibility to Social Influences; IIB: Impulse Buying Tendency; CR: Cognitive Reactions; AR: Affective Reactions; UBI: Urge to Buy Impulsively; SP: Scarcity Persuasion; PP: Price Perception; IBB: Impulse Buying Behavior.

**Source:** Author's data analysis.

### 4.3 Hypothesis testing

This research also assessed the explanatory power of the structural model. Following Hair et al. [92], this study used the coefficient of determination (R^2^) to assess the model's explanatory power, with a value range for R^2^ of 0–1, with 0.75, 0.5, and 0.25 indicating substantial, moderate, and weak, respectively. The structural model results in Table 6 present moderate explanatory power for the UBI (R^2^ = 0.498), indicating the 49.8% of the variance in the UBI can be explained by the exogenous constructs linked to it in this study, including SSI, IIB, CR, and AR. The explanatory power of IBB (R^2^ = 0.445) was also moderate, meaning the 44.5% of its variance can be explained by the UBI. Thus, the outcomes provide strong evidence for our proposed structural model.

**Table 6:** Hypothesis testing.

Hypothesis		Beta	Confidence Intervals	T Value	R^2^	f^2^	Decision
Factors affecting of the urge to buy impulsively							
H_1_	SSI → UBI	0.226	(0.166, 0.286)	6.210***		0.074	Supported
H_2_	IIB → UBI	0.279	(0.218, 0.340)	7.609***	0.498	0.118	Supported
H_3_	CR → UBI	0.196	(0.140, 0.253)	5.660***		0.055	Supported
H_4_	AR → UBI	0.265	(0.207, 0.323)	7.529***		0.111	Supported
*Factors affecting impulse buying behavior during livestreaming*							
H_5_	UBI → IBB	0.332	(0.273, 0.390)	9.320***	0.445	0.150	Supported
*Mediating effect of the urge to buy impulsively*							
H_6_	SSI →UBI →IBB	0.075	(0.051, 0.101)	5.008***			Supported
H_7_	IIB →UBI →IBB	0.093	(0.067, 0.120)	5.806***			Supported
H_8_	CR →UBI →IBB	0.065	(0.044, 0.088)	4.959***			Supported
H_9_	AR →UBI →IBB	0.088	(0.065, 0.113)	5.990***			Supported
*Moderating effects of price perception and scarcity persuasion*							
H_10_	PP x UBI →IBB	0.142	(0.090, 0.193)	4.532***		0.020	Moderates
H_11_	SP x UBI → IBB	0.111	(0.060, 0.163)	3.553***		0.032	Moderates

**Notes:** *** indicates the 1% level of significance. SSI: Susceptibility to Social Influences; IIB: Impulse Buying Tendency; CR: Cognitive Reactions; AR: Affective Reactions; UBI: Urge to Buy Impulsively; SP: Scarcity Persuasion; PP: Price Perception; IBB: Impulse Buying Behavior.

**Source:** Author's data analysis.

**Fig. 1:**
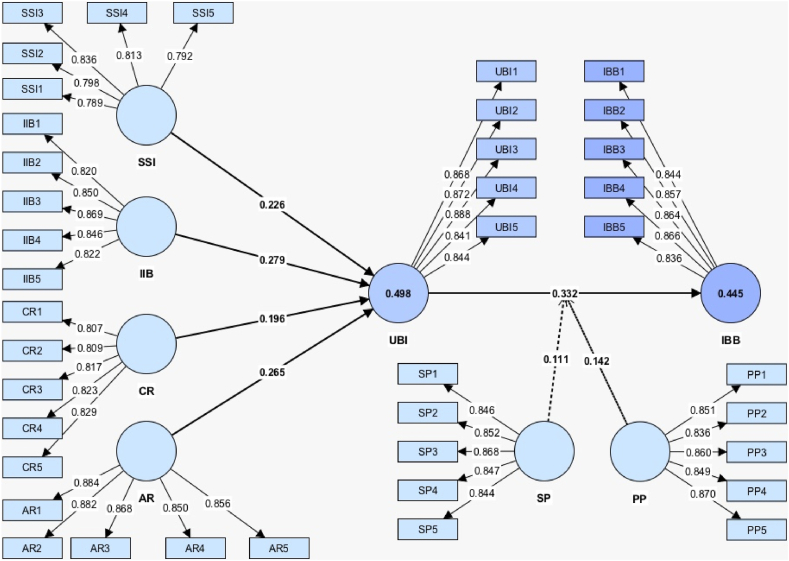
Research framework.

After assessing the measurements, this study evaluated the structural model. First, the structural model's collinearity was evaluated. According to the recommendations of Hair et al. [93], this study used VIF to further validate collinearity. As per Table 3, the results ranged from 1.256 to 1.687, which is less than 3. This indicates with certainty that no multicollinearity problems were present in this research [94]. The next step was to assess the relationship and significance amount of the constructs related to IBB. In terms of path coefficients, this study proposed 11 hypotheses, and the results of hypothesis testing are shown in Table 6 and Fig. 1. The result demonstrated that SSI (β = 0.226, t = 6.210, p < 0.01), IIB (β = 0.279, t = 7.609, p < 0.01), CR (β = 0.196, t = 5.660, p < 0.01), and AR (β = 0.265, t = 7.529, p < 0.01) all had significant positive connections with the UBI. Thus, H_1_, H_2_, H_3_, and H_4_ were supported. Moreover, the urge to buy impulsively (β = 0.332, t = 9.320, p < 0.01) had a significant positive link with impulse buying behavior; thus, H_5_ was supported.

The outcomes of testing the mediating effects indicated that the UBI have a positive mediation effect on the relationships between SSI (β = 0.075, t = 5.008, p < 0.01), IIB (β = 0.093, t = 5.806, p < 0.01), CR (β = 0.065, t = 4.459, p < 0.01), and AR (β = 0.088, t = 5.990, p < 0.01) and IBB. Thus, H_6_, H_7_, H_8_, and H_9_ were supported.

**Fig. 2:**
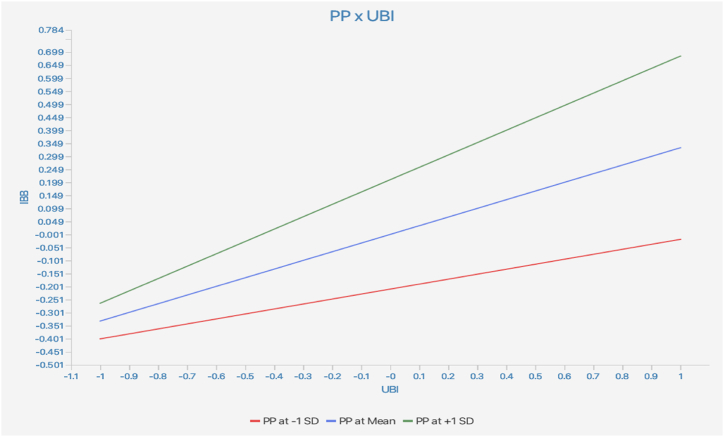
Moderating effect of price perception.

**Fig. 3:**
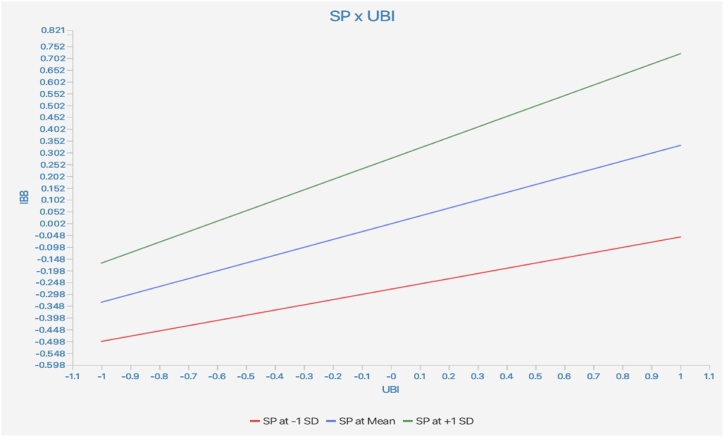
Moderating effect of scarcity persuasion.

Moreover, the results of testing the moderating effects, as shown in Table 6 and Fig. 2, Fig. 3 indicated, that price perception (β = 0.142, t = 4.532, p < 0.01) and scarcity persuasion (β = 0.111, t = 3.553, p < 0.01) significantly moderated the connection between the UBI and IBB. Therefore, H_10_ and H_11_ were supported.

## 5 Discussion

To provide deeper insight into the IBB of Chinese customers during livestreaming, this study examined the determinants of online IBB during livestreaming. The research formulated a theoretical model based on the S–*O*-R model adopted from the existing literature. The data analysis outcomes demonstrated that all the hypotheses proposed in this research were supported. Further, the results offered strong evidence that the structural model proposed in this research has good predictive power. The detail findings are discussed below.

First, in terms of direct effects, SSI showed a positive relationship with the urge to buy impulsively. The results provide strong evidence that susceptibility to social influence positively and strongly influences the UBI. If individuals are likely to rely on the approval of social groups or others to make decisions, then they will be more likely to have a high UBI. This finding is in line with previous work by other researchers [24,44], who also reported a positive and statistically significant relationship between SSI and the UBI. Also, this result aligns with existing theories in consumer psychology, such as social influence theory, which posits that individuals are influenced by the actions and opinions of others, especially in online social contexts. Livestreaming platforms often facilitate interactions between viewers and influencers, creating a social environment where purchasing decisions can be influenced by peer behavior. Thus, it underscores the impact of social factors, such as peer recommendations or social validation, on impulsive buying tendencies in the digital marketplace.

Similarly, individual IIB was found to significantly positively predict individual UBI. These findings correspond to previous studies in consumer behavior [25,48,49], highlighting the importance of individual traits like impulsivity in shaping impulsive buying choices. Consumers are more inclined to make impulsive purchases when they have higher IIB. Hence, in the context of Chinese consumers, this study found that, during livestreaming, higher tendencies toward making impulsive purchases trigger an urge to make an instant purchase. Livestreaming platforms, known for their dynamic and interactive features, could amplify impulsive inclinations by fostering a feeling of urgency and thrill. Consequently, individuals prone to impulsive buying may find themselves more drawn to spontaneous purchases while immersed in livestreaming content.

This finding resonates with existing research [32,53,55] in consumer psychology, highlighting the cognitive processes involved in impulsive buying behavior. Therefore, Chinese customers would be more likely to experience an UBI if livestreaming platforms could attract their attention by offering precise information that would be beneficial to them. Livestreaming platforms, with their immersive and interactive nature, may trigger cognitive reactions such as heightened attention, information processing, and decision-making, which in turn contribute to an increased urge to buy impulsively. Strategies that stimulate cognitive engagement, such as providing detailed product information or interactive product demonstrations, may enhance consumers' urge to make impulsive purchases.

This study's results also indicated that affective reactions are a statistically significant factor of the UBI among Chinese consumers during livestreaming. These findings are in line with Verhagen and van Dolen [57], Ning Shen, & Khalifa [58], and Xiang et al. [40], who also demonstrated a positive link between AR and the UBI. Hence, this study's findings suggest that the UBI can be enhanced if a livestream is exciting, enthusiastic, creative, and inspiring, which can lead consumers to experience positive emotions. Livestreaming platforms, with their dynamic and interactive nature, can evoke strong emotional responses such as excitement, anticipation, and desire, which may heighten individuals' urges to engage in impulsive buying behaviors. Strategies that evoke positive emotions and emotional engagement, such as using emotive language, storytelling, or incorporating user-generated content, may effectively stimulate consumers' urges to make impulsive purchases.

The Hypothesis (H5) delves into the relationship between the UBI and IBB during livestreaming e-commerce. The substantial positive beta coefficient of 0.332 suggests a significant and strong association between these variables, indicating that individuals experiencing a heightened urge to make impulsive purchases are more likely to engage in actual impulsive buying behavior while participating in livestreaming activities. This finding is coherent with existing theories in consumer psychology [33], particularly the notion that the urge to buy impulsively serves as a precursor to actual impulsive buying actions. Livestreaming platforms, with their immersive and interactive features, create an environment conducive to impulsive behavior, where individuals may act on their urges to make spontaneous purchases in real-time. Initiatives that amplify and channel consumers' urges towards impulsive purchases, such as limited-time offers, exclusive deals, or interactive purchase prompts, may effectively boost sales and revenue within this digital marketplace.

The Hypothesis H6 to H9 investigate the mediating effect of the UBI on the relationships between various psychological and emotional factors (SSI, IIB, CR, AR) and IBB within the context of livestreaming e-commerce. This has also been demonstrated in past researches [24,26,30,31,53]. These hypotheses propose that the urge to buy impulsively serves as a mediator, transmitting the influence of psychological and emotional factors to impulsive buying behavior. Also, this indicates that these psychological and emotional factors not only directly influence impulsive buying behavior but also indirectly through their impact on individuals' urges to make impulsive purchases during livestreaming activities. This finding underscores the multifaceted nature of impulsive buying behavior and highlights the critical role played by the urge to buy impulsively in translating various cognitive and emotional factors into actual purchasing decisions. Livestreaming platforms, with their real-time and interactive features, may amplify the influence of these psychological and emotional factors on impulsive buying behavior, making the mediation effect of the urge to buy impulsively particularly salient in this context. Understanding the mediating role of the urge to buy impulsively can inform marketers and platform operators about the underlying mechanisms driving impulsive buying behavior in livestreaming e-commerce.

Lastly, hypothesis H10 and H11 explore the moderating effects of PP and SP on the relationship between the UBI and IBB within the context of livestreaming e-commerce. These hypotheses propose that the effects of the urge to buy impulsively on impulse buying behavior may vary depending on individuals' perceptions of price and susceptibility to scarcity persuasion. The significant positive beta coefficients suggest that both price perception and scarcity persuasion indeed moderate the relationship between the urge to buy impulsively and impulse buying behavior, which is consistent with extant literature related to purchase behavior [65]. This indicates that individuals' perceptions of price and susceptibility to scarcity persuasion influence the extent to which their urge to make impulsive purchases translates into actual buying behavior during livestreaming activities. These findings underscore the importance of external factors in shaping impulsive buying behavior in livestreaming e-commerce. Price perception and scarcity persuasion strategies employed by marketers and platform operators can significantly impact consumers' impulsive buying tendencies and subsequent purchasing decisions.

## 6 Conclusion, implications and limitations

### 6.1 Conclusions

This research evaluated the predictors that affect online IBB during livestreaming to offer a deeper comprehension of the IBB of Chinese consumers. The study developed a theoretical framework based on the S–*O*-R model, and the findings indicate that SSI, impulse buying tendency, CR, and AR have statistically significant effects that can trigger the UBI during a livestream among Chinese consumers. Further, this research identified that the UBI strongly mediates the relationship between SSI, IIB, CR, AR, and IBB. In addition, the study showed that PP and SP significantly moderate the UBI and IBB.

### 6.2 Implications

#### 6.2.1 Theoretical implications

This research makes several theoretical contributions to the prevailing literature of online impulsive buying during livestreaming. *First,* by using S–*O*-R theory, this research broadens the research horizon of this theory when applied to impulse buying in livestreaming e-commerce, particularly in the Chinese context, which enriches the knowledge on related theories and context of livestreaming impulse buying.

*Second,* this study extended the S–*O*-R theory and its application in livestreaming context considering scarcity persuasion, price perception SSI, IIB, CR, and AR, which have received less attention in previous studies [6,19]. Hence, this study added new information pertaining to susceptibility to social influence, individual impulse buying tendency, cognitive reactions, and affective reactions, as it found that these variables are significant predictors of impulse buying behavior during livestreaming under the theory of S–*O*-R.

*Third,* the results validate the multifaceted nature of impulsive buying behavior. The significant associations between SSI, IIB, CR, AR, and the UBI suggest that multiple psychological and emotional factors converge to drive the impulsive purchasing inclination of consumers in the digital marketplace. This supports the notion that consumer behavior is a complex interplay of various cognitive and emotional processes.

*Fourth*, this study adds to the existing body of literature by providing insights into impulse buying within the realm of livestreaming e-commerce by incorporating scarcity persuasion and price perception as moderators, particularly in the Chinese context, which provides deeper insight into the external factors that may influence whether consumers make impulse purchases. Hence, the findings add new insight to the existing knowledge about scarcity persuasion, as prior studies have found that PP [95] and SP [65] are direct predictors of IBB.

*Fifth*, the mediating effect of the UBI on the relationships between different psychological and emotional factors and IBB underscores the role of UBI as a critical psychological mechanism. This finding emphasizes that the desire or urge to make an impulsive purchase plays a crucial role in translating various cognitive and emotional factors into actual buying decisions. This insight contributes to a greater nuanced comprehension of the cognitive processes involved in impulsive buying.

#### 6.2.2 Practical implications

Regarding the practical implications, *first,* livestreaming e-commerce is a comparatively novel field that is expected to show immense growth and revolutionize how consumers shop. Therefore, this study's findings are important and a significantly helpful source of valuable information for the leading Chinese market players, as well as new entrants, who want to develop new strategies for the future and strengthen their position in the livestreaming market. By tailoring marketing strategies to resonate with specific psychological and emotional triggers, companies can engage consumers in a more personalized manner, increasing the likelihood of impulse purchases.

*Second*, this study's finding showed that susceptibility to social influence, individual impulse buying tendency, cognitive reactions, and affective reactions are important predictors of the urge to make an online impulse purchase for consumers over the age of 18 in various locations across China. Hence, e-commerce livestreaming sellers should be aware that, to entice Chinese consumers to make immediate purchases during livestreaming, they should concentrate on the susceptibility to social influence factor by encouraging other customers to leave online reviews and recommend the items to family and friends. Similar to how individual impulse buying tendency was revealed to be a statistically significant factor of the UBI, a tendency toward impulse buying needs to be fostered among Chinese customers.

*Third*, this study also suggests that cognitive and affective reactions are statistically significant for generating the UBI. Therefore, vendors who use e-commerce livestreaming should put more effort toward perceived enjoyment and usefulness of livestreaming shopping by improving the quality of their livestreaming system. Recognizing the influence of psychological and emotional factors on consumer behavior, businesses can engage in educational initiatives. By sharing information about cognitive biases, emotional triggers, and decision-making processes, brands can empower consumers to make more informed choices, potentially leading to more deliberate purchasing decisions.

*Fourth,* the findings emphasize the importance of a seamless and user-friendly online shopping experience. Businesses should prioritize user interface design, navigation ease, and checkout processes to reduce friction in the purchasing journey. A streamlined experience aligns with the rapid decision-making associated with impulsive buying, encouraging consumers to complete purchases without unnecessary hurdles. Moreover, employing data analytics and consumer behavior insights can guide businesses in crafting personalized recommendations and product offerings. By analyzing consumers' historical buying patterns and preferences, companies can suggest products aligned with individual impulsive tendencies, enhancing the probability of impulse purchases.

*Fifth,* recognizing the amplified impact of the UBI on IBB during livestreaming activities presents an opportunity for businesses to leverage these platforms strategically. Brands can enhance consumer engagement during livestreaming sessions by integrating interactive features that capitalize on the immediacy and dynamic nature of the medium, thereby encouraging impulsive buying behaviors.

*Sixth,* acknowledging the mediating effect of UBI between various psychological factors and IBB underscores the importance of incorporating psychological frameworks into marketing strategies. By understanding how cognitive and emotional factors contribute to the UBI, companies can create content and messaging that resonate with these psychological triggers, encouraging more conversions. Platform users should ensure their platform's design and features facilitate UBI. This includes providing easy access to product information, customer reviews, and interactive features that trigger impulsive buying decisions.

*Seventh,* the moderating effects of Price Perception and Scarcity Persuasion on the relationship between UBI and IBB offer actionable insights into leveraging external cues. Businesses can employ effective pricing strategies and scarcity messaging to influence consumer perceptions and amplify the impact of impulsive tendencies. Limited-time offers, flash sales, and scarcity-driven promotions can capitalize on consumers' heightened urge to make impulsive purchases.

### 6.3 Limitations and future research directions

While this study offers notable contributions, there are inherent limitations that warrant attention in future research endeavors. *First,* as the research was specifically designed to study Chinese consumers who are involved in online purchases during livestreaming, the findings may be difficult to generalize to other countries or regions. Therefore, further studies could extend the research to other countries or an international context**.** *Second,* regarding the research design, this study collected data from different participants at a single timepoint; however, the findings could vary over time. Thus, future studies should investigate the online impulse buying phenomenon during livestreaming through longitudinal research to provide new insights. Third, to simplify the model, this study omitted the direct relationship between price perception and UBI and IBB. Additionally, price discounts and coupons can be included as moderating factors in the framework. Future studies can explore these relationships and constructs to create a more comprehensive framework.

## Ethics approval

The Human Resource Ethics Committee and the Executive Committee of School of commercial, Nantong Institute of Technology approved this study (Reference number: BS-NIT-2023-0403) dated April 17, 2023.

## Informed consent to participate

Informed consent for participation was obtained from respondents who participated in the survey. No data was collected from anyone under 18 years old.

## Consent to publish

All authors approved the manuscript and give their consent for submission and publication.

## Funding

This study is supported via funding from 10.13039/501100002703Jiangsu University Philosophy and Social Science Research Project (Project Number: 2023SJYB1708).

## Availability of data and materials

The data is available in the supplementary documents within this article.

## CRediT authorship contribution statement

**Zhitan Feng:** Writing – original draft, Methodology, Investigation, Conceptualization. **Abdullah Al Mamun:** Writing – review & editing, Methodology, Formal analysis, Conceptualization. **Mohammad Masukujjaman:** Writing – review & editing, Methodology, Formal analysis, Conceptualization. **Mengling Wu:** Writing – original draft, Methodology, Investigation, Conceptualization. **Qing Yang:** Writing – original draft, Methodology, Investigation, Conceptualization.

## Declaration of competing interest

The authors declare that they have no known competing financial interests or personal relationships that could have appeared to influence the work reported in this paper.
