# Supplementary material for: Impulse buying behavior during livestreaming: Moderating effects of scarcity persuasion and price perception
Source: Heliyon. 2024 Mar 18;10(7):e28347. doi: 10.1016/j.heliyon.2024.e28347 (PMC10979273; doi:10.1016/j.heliyon.2024.e28347)
Supplement: Multimedia component 1 [file mmc1.docx]

**Supporting Material - S1**. Survey Instrument

| Code | Questions | |
| --- | --- | --- |
| SSI1 | In the live stream, I usually buy products that I think others will recognize. | |
| SSI2 | If other people can see that I'm using a product, I usually buy what they want me to buy. | |
| SSI3 | In the live stream, I gain a sense of belonging by purchasing the same products or brands that others have purchased. | |
| SSI4 | If I want to be like someone, I often try to buy the same products that they do. | |
| SSI5 | In the live stream, I often identify with other people by purchasing the same products and brands they do. | |
| IIB1 | I often buy things spontaneously. | |
| IIB2 | I often buy things without thinking. | |
| IIB3 | "I see it, I buy it" describes my shopping behaviour. | |
| IIB4 | "Buy now, think about it later" describes my shopping behaviour. | |
| IIB5 | Sometimes I feel like buying things on the spur of the moment. | |
| CR1 | The live streaming platform piqued my curiosity and facilitated problem-solving. | |
| CR2 | During live streaming, I need accurate product information. | |
| CR3 | I want to know the user’s evaluation of the live streaming platform products. | |
| CR4 | The products of this live streaming platform provided me with the necessary information. | |
| CR5 | The products of this live streaming platform draw attention to new information. | |
| AR1 | Live streaming is exciting. | |
| AR2 | Live streaming is enthusiastic. | |
| AR3 | Live streaming is inspiring. | |
| AR4 | Live streaming is really unusual. | |
| AR5 | Live streaming is very attractive. | |
| UBI1 | When I watched the live streaming, I felt an impulse to buy items other than my specific shopping goals. | |
| UBI2 | When I watch the live streaming, I want to buy unplanned items that do not meet my specific shopping goals. | |
| UBI3 | When I watch the live streaming, I tend to buy unplanned items that exceed my specific shopping goals. | |
| UBI4 | When I see something I like, I buy it. | |
| UBI5 | Now is the time to buy; later will be the time to ponder about it. This is how I shop. | |
| SP1 | I think the current supply of this live streaming platform is very small. | |
| SP2 | I think the products on this live streaming platform will soon be sold out. | |
| SP3 | I think many people will buy this live product. | |
| SP4 | I think the limited sales of live streaming platform products will make many people buy them. | |
| SP5 | I think the shopping time provided by the live streaming platform is very tight. | |
| PP1 | This live streaming platform provides the best possible price to meet my needs. | |
| PP2 | I am not willing to make extra effort to find a lower price. | |
| PP3 | The price of product delivery corresponds to its performance. | |
| PP4 | The discount price on the live streaming platform is very cheap. | |
| PP5 | The price of products on this live streaming platform is reasonable. | |
| IBB1 | | When I bought products online, I felt an unprompted urge to buy them. |
| IBB2 | | Without intending to, I ended up purchasing the thing. |
| IBB3 | | I bought this on the spur of the moment. |
| IBB4 | | I bought it rashly. |
| IBB5 | | My definition is "If I see it, I will buy it." |

**Notes:** SSI: Susceptibility of Social Influences; IIB: Impulsive Buying Tendency; CR: Cognitive Reactions; AR: Affective Reactions; UBI: Urge to Buy Impulsively; SP: Scarcity Persuasion; PP: Price Perception; IBB: Impulse Buying Behaviour.
